# A single intranasal dose of essential oil spray confers modulation of the nasopharyngeal microbiota and short-term inhibition of *Mannheimia* in feedlot cattle: a pilot study

**DOI:** 10.1038/s41598-023-50704-1

**Published:** 2024-01-08

**Authors:** Gabriela Magossi, Kaycie N. Schmidt, Thomas M. Winders, Zachary E. Carlson, Devin B. Holman, Sarah R. Underdahl, Kendall C. Swanson, Samat Amat

**Affiliations:** 1 Department of Microbiological Sciences, North Dakota State University, Fargo, ND 58108 USA; 2 Lacombe Research and Development Centre, Agriculture and Agri-Food Canada, 6000 C & E Trail, Lacombe, AB T4L 1W1 Canada; 3 Department of Animal Sciences, North Dakota State University, Fargo, ND 58102 USA

**Keywords:** Microbiology, Respiratory tract diseases

## Abstract

Five essential oils (EOs) were previously characterized in vitro and identified as candidate EOs for the development of an intranasal EO spray to mitigate bovine respiratory disease (BRD) pathogens. In the present study, these EOs were evaluated for their potential to (i) reduce BRD pathogens, (ii) modulate nasopharyngeal microbiota, and (iii) influence animal performance, feeding behavior and immune response when a single dose administered intranasally to feedlot cattle. Forty beef steer calves (7–8 months old, Initial body weight = 284 ± 5 kg [SE]) received either an intranasal EO spray (ajowan, thyme, fennel, cinnamon leaf, and citronella) or PBS (Control; n = 20/group) on day 0. Deep nasopharyngeal swabs were collected on days (d) -1, 1, 2, 7, 14, 28, and 42 and processed for 16S rRNA gene sequencing, qPCR, and culturing. Significant effects of EO on community structure (d1), microbial richness and diversity, relative abundance of some dominant phyla (d1, d2, and d14), and the overall interaction network structure of the nasopharyngeal microbiota were detected. The relative abundance of *Mannheimia* was lower in the EO calves (4.34%) than in Control calves (10.4%) on d2, and *M. haemolytica* prevalence on d7 as compared to control calves. Feed intake, average daily gain, feeding behavior, and blood cell counts were not affected by EO treatment. Overall, a single intranasal dose of EO spray resulted in moderate modulation of nasopharyngeal microbiota and short-term inhibition of *Mannheimia* while not influencing animal performance, feeding behavior or immune response. Our study, for the first time, shows the potential use of intranasal EO to mitigate BRD in feedlot cattle.

## Introduction

Bovine respiratory disease (BRD) is one of the most significant health problems affecting both the dairy and beef cattle industries in the U.S., accounting for losses of more than $900 million due to reduced performance and cattle death^1^. Feedlot cattle are the most affected by BRD. Calves entering the feedlot are often exposed to a number of stressors including weaning, transportation, and comingling at auction markets^2^. As a result, calves experience suppressed immunity, viral infections, and/or disrupted respiratory microbiota homeostasis. This ultimately predisposes the host to allow for the rapid growth of pathogenic bacteria in the upper respiratory tract, which then translocate into the lungs, which can result in the development of pneumonia^2,3^. To combat BRD, cattle producers often adopt metaphylaxis practices that treat every animal that enters the feedlot with antibiotics, regardless of health status. The primary prevention/treatment of BRD in feedlot cattle is the mass application of broad-spectrum antibiotics, mainly macrolides (tulathromycin, tilmicosin, gamithromycin, tildipirosin, and tylosin) and fenicols (florfenicol), but other drug classes are also approved for use in the prevention and treatment of BRD in feedlot cattle, like tetracyclines (tetracycline, chlortetracycline, oxytetracycline), fluorquinilones (danofloxacin, enrofloxacin), sulfonamides (sulfamidine), cephalosporins (ceftiofur)^4,5^. Animals are also vaccinated against the viral pathogens associated with BRD. Resistance to enrofloxacin, florfenicol, and macrolides detected in BRD pathogens after metaphylactic treatment increases drastically (0–3% to 68–99% of isolates) when compared to bacteria that were not exposed to antibiotics^6^. Despite the use of antibiotic metaphylaxis upon feedlot arrival and current treatment protocols, the number of cattle developing BRD continues to rise due to an increase in antibiotic-resistant pathogens^7^. Recent studies conducted in commercial feedlots across North America^6,8,9^ showed high levels of resistance (≥ 50%) in respiratory pathogens isolated from feedlot cattle, with some bacteria resistant against multiple classes of antibiotics. As high levels of resistance to antimicrobials are emerging in feedlots, the development of novel antimicrobial agents to control pathogenesis is needed.

Modulation of the bovine respiratory microbiota may provide an alternative opportunity to enhance colonization resistance against BRD pathogenic agents including multidrug-resistant pathogens^3,10,11^. A diverse and dynamic microbial community resides within the respiratory tract that contributes to pulmonary health by stimulating the immune response and providing colonization resistance against pathogens. Disruption of the respiratory microbiome resulting from typical management practices, which include weaning, transportation, feed transition, and antibiotic administration, can compromise microbiome-mediated mucosal defense against infectious threats^12^. Thus, restoring homeostasis of the respiratory microbiota by selectively inhibiting pathogenic bacteria while promoting beneficial endogenous members holds the potential for mitigating BRD^13^.

Essential oils (EOs) are natural plant extracts that are usually obtained by a distillation process and are composed of terpenes and volatile compounds which have antimicrobial properties^14^ and are thought to have the potential to enhance microbiome-mediated respiratory health^15^. The EO extracted from aromatic and medicinal plants can inhibit various bacterial pathogens associated with human respiratory infections^16–18^. Thus, the potential application of EO as a nasal spray to mitigate respiratory infections has been tested in human patients suffering from upper respiratory tract infections without any adverse effects on the respiratory tract^19–21^. Research indicates that the application of EO in conjunction with antibiotics can have an impact on antimicrobial resistance (AMR). Specifically, studies have demonstrated that when EO is used in combination with antibiotics against multidrug-resistant bacteria, these organisms regain susceptibility to antibiotics^18,22–24^. Interestingly, however, EOs displayed limited antibacterial activity against beneficial bacteria such as lactic acid-producing bacteria^25–27^. Furthermore, antiviral activity of EOs against respiratory viral pathogens^28^, and immunomodulatory activities of EOs have been documented^29^.

The antibacterial and antiviral activities of EOs against human respiratory pathogens, their limited effects on beneficial bacteria, and their immunomodulatory activity, suggests that EO may also be effective in mitigating BRD in feedlot cattle. Major EO compounds such as carvacrol and thymol have shown potential as antibiotic adjuvants against BRD-associated bacterial pathogens^15,30^. Moreover, we have recently published data showing that EOs inhibit the BRD bacterial pathogens *Mannheimia haemolytica*, *Pasteurella multocida*, and *Histophilus somni *in vitro^31,32^. The EOs of ajowan, thyme, and cinnamon leaf completely or partially inhibited these BRD pathogens when applied in a vapor phase. When tested in liquid phase, the ajowan, thyme, and fennel most effectively inhibited all three BRD pathogens tested including multidrug-resistant strains with minimum inhibitory concentrations (MICs) of ≤ 0.025% (v/v) for each EO. For these EO, the MIC was 2–32 fold greater for commensal bacteria (i.e., *Lactobacillus* spp., *Bacillus* spp. and *Staphylococcus* spp.) compared to BRD-associated pathogens.

No cytotoxic effects of EOs against bovine turbinate (BT) cells were observed within the tested range of concentrations (≤ 0.4%, v/v)^31^. Thus, EOs are less likely to have negative effects on the commensal bacterial community within the bovine respiratory tract when they are administered to target pathogens. Recently, we further evaluated the selected EOs for their antimicrobial, antiviral, immunomodulatory, and antibiofilm activities, as well as their effect on the culturable fraction of the bovine nasopharyngeal microbiota in vitro^33^. Based on these screening criteria, we identified five EOs as strong candidates for the development of an intranasal EO spray to mitigate bovine respiratory disease pathogens in beef cattle. The main objective of this pilot study was to further evaluate the effects of a single intranasal dose of selected EOs on the nasopharyngeal bacterial microbiota, complete blood cell count (CBC), and animal performance in beef steer calves.

## Materials and methods

All animal procedures conducted in this study were reviewed and approved by the North Dakota State University Institutional Animal Care and Use Committee (protocol ID: I ACUC20210060). We confirm that all methods were carried out in accordance with relevant guidelines including ARRVE guidelines and regulations.

### Animal husbandry and experimental design

A total of 79 crossbred Angus steer calves from the North Dakota State University (NDSU, Fargo, ND, USA) beef cattle herd that were born between April and June of 2021 were used in this study. These calves were raised with their dams on pasture of the Ekre Grassland Preserve southwest (Kindred, ND, USA) until weaning. At weaning, calves were transported (about 65 km distance) to the NDSU main beef cattle facility, where they were separated from their dams and weaned. After weaning, calves were chosen and transported to (2 km distance) the NDSU Beef Cattle Research Complex (BCRC) where they were housed in four partially covered pens. All calves were individually fed with the Instentec BV feeding system (Hokofarm Group, Marknesse, the Netherlands) which was on a cement pad. The calves were trained to use the Instentec system for at least four weeks. After the training period, seven calves that failed to train to use the Calan gates were removed from the study. The remaining steers were individually fed two different finishing diets consisting of 20% corn silage, 0% (diet 1) or 20% (diet 2) dried distiller of grains solubles (DDGS), 75% (diet 1) or 55% (diet 2) corn grain, and 5% supplement (dry matter basis) (n = 36 head/diet). On d0, 20 calves within each of the 2 dietary groups were randomly assigned either to the EO group (n = 20), or the control group (n = 20). The EO group received an intranasal EO blend spray in sterile phosphate-buffered saline (PBS; Corning, Corning, NY, USA), while the control group received an intranasal spray containing PBS without any EO. Of note, all calves were sampled using deep nasopharyngeal swabs, as previously described^34^, to evaluate their *M. haemolytica* status (i.e., positive or negative) on d-1, and *M. haemolytica* culture results were used to equally distribute the *M. haemolytica*-positive calves between the EO or control groups. This animal trial was conducted between Dec 2021 and Feb 2022.

### Preparation of the essential oil spray

Based on our previous in vitro screening and characterization of 15 commercial EO^31,32^, and recent evaluation of the selected EOs for their antimicrobial, antiviral, immunomodulatory and antibiofilm activities, as well as their effect on bacterial isolates from the bovine nasopharyngeal tract in vitro^35^, five EOs (ajowan, thyme, fennel, citronella, and cinnamon leaf) were selected for inclusion in the EO spray. About 16 h prior to the intranasal administration of the EO spray to the calves, the EO spray mixture was prepared. Briefly, 25 µl of each EO was diluted in 975 µl dimethyl sulfoxide (DMSO, Sigma-Aldrich, St. Louis, MO). Then, each of the EO stock solutions were further diluted with PBS (pH 7.4) and then mixed to a final concentration of 0.025% (v/v) each in PBS. The final concentration of DMSO in the EO blend was 1.125% (v/v). The control spray contained PBS with DMSO (1.125%) but no EO. Three milliliters of EO blend were loaded into a sterile 10 ml syringe covered with a sterile needle to prevent leakage. For the control group, 3 ml PBS was loaded similarly into a syringe.

### Administration of EO spray

On d0, calves were restrained in a squeeze chute and administered treatments. The head was held by personnel to prevent movement, and the nostrils were wiped clean with a paper towel sprayed with 70% ethanol. Then, sterile laryngo-tracheal mucosal atomization devices (LMA MADgic Laryngo-Tracheal Mucosal Atomization Device without syringe, Cat# MAD 700, Teleflex, Morrisville, NC, USA) were fitted to the EO- or PBS-loaded syringes, and the atomization device was inserted into each nostril (approximately 15 cm) and injected until the syringe was empty. One atomization device was used for the two nostrils of each calf. A total of 6 ml of EO spray (3 ml per nasal cavity) was administered to calves in the EO treatment group. For the control group calves, PBS was sprayed into the nostrils similar to the EO inoculum (3 ml per nostril).

### Animal growth performance, feeding behavior, and health monitoring

**Figure 1:**
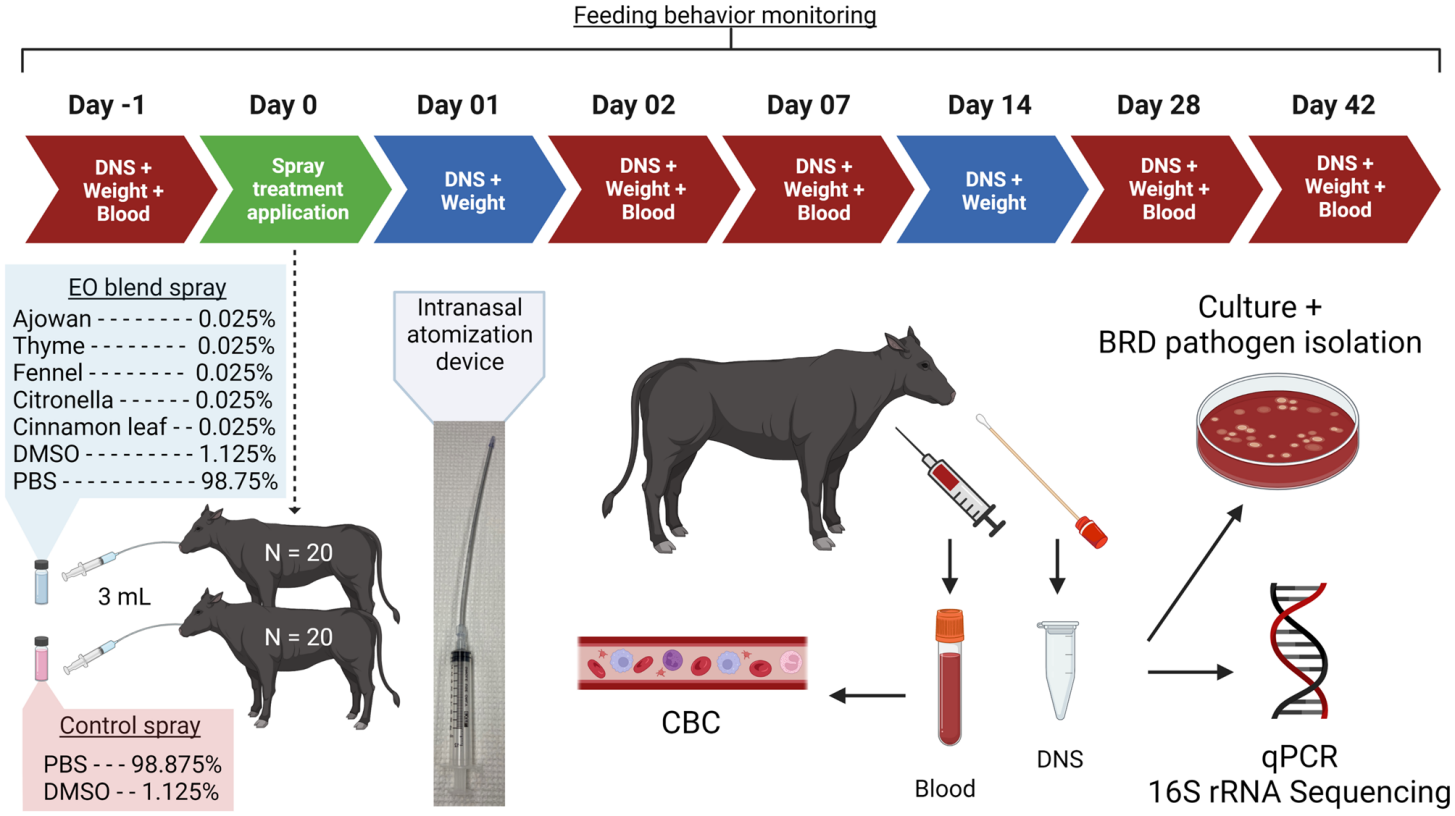
Experimental design, sample collection, and analyses. Figure was created with BioRender.com.

During the 42-day study period, calves were weighed on days (d) − 1, 0, 1, 2, 7, 14, 28, and 42, and their daily feed intake and feeding behavior were recorded (Fig. 1). All animals were brought to a mechanical chute for weighing, sample collection, and visual assessment for symptoms indicative of BRD in the morning. These assessments included abnormal respiratory rate, and nasal and ocular discharge. Feeding behavior was measured daily using the Insentec BV feeding system based on the duration and number of times that an animal used the feed bunk, in addition to the amount of feed ingested determined by a difference in weight before and after each visit. Visits were determined by the approach and use of the feed bunk, and meals are defined by combining visits which are < 7 min apart from each other.

### Nasopharyngeal swab sampling and processing

A deep nasopharyngeal swab (DNS) from the right nostril of each calf was collected on day (d) − 1 (24 h prior to EO administration) and 1, 2, 7, 14, 28 and 42 days after intranasal EO administration (Fig. 1). An extended guarded swab (27 cm) with a rayon bud (MW 124, Medical Wire & Equipment, Corsham, England) was used for DNS sampling as previously described^36^. Briefly, prior to sampling, the right nostril of the calf was wiped clean with 70% ethanol and a paper towel. The extended guarded swab was passed into the nostril and when the sheathed swab reached the nasopharynx area, the swab tip was advanced a few centimeters into the nasopharynx and rotated. The swab was withdrawn into the sheath and removed from the nasal cavity. The swab tip (approx. 2.5 cm) was then snipped, transferred to a sterile microfuge tube using a sterilized wire cutter and then transported to the lab on ice. Upon arrival in the lab, the nasopharyngeal swabs were transferred into 1 ml of brain heart infusion (BHI) broth (BD, Franklin Lake, NJ, USA) containing 20% glycerol. A 100 µl aliquot was used for culturing and the remainder stored at − 80 °C until DNA extraction.

### Genomic DNA extraction, 16S rRNA gene sequencing and analysis

Genomic DNA was extracted from all of the DNS samples (d-1, 1, 2, 7, 14, 28, and 42) using the Qiagen DNeasy Blood and Tissue kit (Qiagen Inc., Germantown, MD, USA) according to the manufacturer’s instructions with some modifications as outlined previously^13,36^. The extracted DNA quality was determined with a Nanodrop spectrophotometer (Thermo Fisher Scientific, Waltham, MA, USA), and concentration assessed using the Quant-iT PicoGreen dsDNA assay kit and a Qubit 4 Fluorometer (Themo Fisher Scientific, Waltham, CA, USA). From the extracted DNA (a total of 266 nasal swabs and 2 control samples), the V3-V4 hypervariable regions of the 16S rRNA gene were amplified using the 341-F (5′- CCTAYGGGRBGCASCAG-3′) and 806-R (5′- GGACTACNNGGGTATCTAAT-3′) primers and sequenced on a NovaSeq 6000 platform (Illumina, San Diego, CA, USA) with a SP flow cell (2 × 250 bp) as previously described ^36^.

The 16S rRNA gene sequences were processed using DADA2 v. 1.20.0^37^ in R. 4.0.3 with the forward reads truncated at 225 bp and the reverse reads at 220 bp and other default parameters (maxN = 0, maxEE = c(2,2), truncQ = 2, rm.phix = TRUE, compress = TRUE, multithread = TRUE). The reads were then merged using mergePairs, sequences shorter than 400 bp in length were discarded, chimeras were removed using the removeBimeraDenovo with the consensus method, and taxonomy was assigned to the resulting amplicon sequence variants (ASVs) using the SILVA SSU release v138.1 database^38^. Next, ASVs that were unclassified or classified as chloroplasts, mitochondria, or eukaryotes were removed, as were samples with fewer than 1000 sequences and singletons. ASVs likely to be contaminants were also removed using the negative extraction controls and the package decontam version 1.16.0^39^. To account for uneven sequence depth, samples were rarefied to even depth (“4577” sequences), prior to the calculation of Bray–Curtis dissimilarities and alpha diversity measures. Alpha and beta diversity, and taxon relative abundance were determined with the phyloseq version. 1.40.0^40^, vegan version 2.6–4, and microbiome version 1.18.0 packages in R studio version 4.2.0. Unless specified, the default parameters were used for the described tools.

### Quantification of total bacterial abundance in nasopharyngeal swab using quantitative PCR

Genomic DNA extracted from the DNS samples was also subjected to quantitative PCR (qPCR) to assess the total 16S rRNA gene copy number, as described previously^33^ to determine the bacterial abundance in each sample. Briefly, the primers 515Fq (5′– GTGYCAGCMGCCGCGGTAA—3′) and 806Rq (5′ -GGACTACNVGGGTWTCTAAT—3′) were used to amplify the V4 region of the of the 16S rRNA gene. The DNA samples were normalized to 25 ng/μl, unless the concentration of was lower than 25 ng/μL, in which case, the DNA was used undiluted. Each qPCR mixture contained 1X SsoAdvanced Universal SYBR Green Supermix (Bio-Rad Laboratories, Inc., Hercules, CA, USA), 0.4 µM (each) primer, 0.1 µg/µl bovine serum albumin (New England Biolabs, Pickering, ON, Canada), 1 μl (25 ng) of DNA template, and 9.4 µl of molecular biology grade water (Corning, Manassas, VA, USA) in a total volume of 25 µl. A CFX96 Touch Real-Time PCR Detection system (Bio-Rad Laboratories) with the following conditions was used: an initial denaturation at 95 °C for 3 min, followed by 40 cycles at 95 °C for 25 s, 50 °C for 30 s, and then 72 °C for 45 s. Standard curves (10^2^–10^8^ gene copies) were generated using the pDrive cloning vector (Qiagen) containing the PCR product from the 16S rRNA gene. All samples for qPCR were performed in duplicate with standards (1 µl) and no-template controls (1 µl of nuclease-free H_2_O), as well as a positive control. A melt curve analysis was performed following qPCR amplification to ensure that only the target gene was amplified. The number of 16S rRNA gene copies per μl was multiplied by the original DNA concentration (25 ng) and the volume (400 μl × 2.5) of nasopharyngeal swab used in DNA extraction, and then the copy numbers were log transformed.

### Culturing, isolation, and detection of bovine respiratory pathogens

Aliquots of swab suspension were plated onto tryptic soy agar (TSA) (BD, Becton Dickinson and Co., Franklin Lakes, NJ, USA) containing 5% sheep blood supplemented with 15 μg/ml of bacitracin (Sigma-Aldrich, St. Louis, MO, USA) to inhibit Gram-positive bacteria. The plates were incubated overnight at 37 °C in 5% CO_2_ (*M. haemolytica* and *P. multocida*) or 10% CO_2_ (*H. somni*). Up to three colonies displaying morphology indicative of *M. haemolytica*, *P. multocida* and *H. somni*^41^ were re-streaked onto TSA with 5% sheep’s blood and incubated overnight at 37 °C in 5% CO_2_. BHI glycerol stocks (1 ml; 20% glycerol) and TE stocks of each BRD pathogen isolate were prepared and immediately stored at − 80 °C (glycerol stock for culturing), and -20 °C (TE stock for further DNA extraction).

Genomic DNA was extracted from a subset of presumptive *M. haemolytica*, *P. multocida* and *H. somni* (N = 104) isolates using a Quick-DNA Fungal/Bacterial Miniprep Kit (Zymo Research, Irvine, CA, USA) according to manufacturer’s instructions with the following modifications: A) 70-µl of TE stock was used as the input material; B) samples were processed at 4.5 m/s for 30 s in a MP FastPrep-24 bead beater (MP Biomedicals, Irvine, CA, USA); and C) DNA was eluted with 40 µl of elution buffer. The extracted DNA was stored at − 20 °C until needed for PCR.

The nearly full-length 16S rRNA gene was amplified via PCR using the primers 27F (5′- AGAGTTTGATCMTGGCTCAG -3′) and 1492R (5′- TACGGYTACCTTGTTACGACTT -3′) as described previously^41,42^. Each PCR consisted of 20 µl iQ Supermix (Bio-Rad Laboratories), 1 µl of each primer (0.25 µM), and 2 µl of isolate DNA for a total volume of 40 µl per reaction. The PCR conditions were as follows: an initial denaturation of 95 °C for 5 min; 35 cycles of 95 °C for 45 s, 50 °C for 30 s, 72 °C for 2 min; and a final extension at 72 °C for 5 min. The PCR were performed using an Eppendorf Mastercycler (Eppendorf, Hamburg, Germany). A 1% (w/v) agarose gel was used to visualize the PCR products and amplicons were sent to MCLAB (San Francisco, CA, USA) for Sanger sequencing. The amplicons were then identified using the Basic Local Alignment Search Tool (BLAST) and the non-redundant NCBI nucleotide database.

### Blood sample collection and analysis

To evaluate if the EO spray induced an inflammatory or immune response, whole blood (5 ml) was collected in the morning at the same time that BW was recorded on days -1, 2, 7, 28 and 42 from the jugular vein using Vacutainer spray coated K2EDTA tubes (Becton Dickinson, Franklin Lakes, NJ, USA). Whole blood samples were then shipped on the same day they were collected to the Texas A&M Veterinary Medical Diagnostic Laboratory (TVMDL, College Station, TX, USA) where a CBC analysis was performed using automated hemocytometer (ADVIA 120, Siemens Healthcare Diagnostics, Tarrytown, NY, USA).

#### Statistical analysis

Body weight (BW), average daily gain (ADG), and average dry matter intake (DMI) were compared between the EO and control groups using either an unpaired two sample t-test or Wilcoxon test depending on the normality of the datasets. The Shapiro-Walk normality test was used to determine whether a dataset followed a normal distribution. Culture data was analyzed with a chi-square model with the culture status (positive or negative) as the binary outcome. The effect of EO treatment on the microbial community structure was evaluated using permutation analysis of variance (PERMANOVA) and Bray–Curtis dissimilarities with the adonis2 function in the vegan package^43^. Alpha diversity metrics including Shannon diversity index, inverse Simpson diversity index and the number of observed ASVs, and microbiome compositional relative abundances were determined using the packages phyloseq v. 1.40.0, vegan v. 2.6–4, and microbiome v. 1.18.0 (https://microbiome.github.io/)^44^.

The number of observed ASVs, Shannon diversity index, and inverse Simpson diversity index were evaluated within individual sampling days using the non-parametric Wilcoxon rank-sum test. The relative abundance of phyla and genera, as well as blood hematology CBC results, and 16S rRNA gene copy numbers were analyzed using a generalized linear mixed model with treatment, sampling day, and the interactions between treatment and sampling day included as fixed effects, and animal as the random effect. Differentially abundant taxa in relation to treatment groups, sampling days, and their interactions were determined with analysis of variance and the multiple comparisons were considered with the Tukey’s and Duncan’s post hoc tests. All analyses were conducted using RStudio version 4.2.2 (http://www.r-project.org/; R Development Core Team, 2015), and a *P* value of less than or equal to 0.05 was considered significant.

Ecological network modeling was performed to evaluate the directed microbial interactions among the nasopharyngeal microbial communities using BEEM-static in R. With all the observed abundance genera profile obtained from all 7 sampling time points, generalized Lotka-Volterra models (gLVMs), using biomass estimation and model inference in an expectation maximization-like algorithm (BEEM), were used to construct two (EO and control groups) ecological network models as described by Li et al.^45^. The interaction network inferred by BEEM-statistic was visualized by using the ggraph package of R. Codes for the analysis can be found at https://github.com/gmagossi/EO_16S.

## Results

### Animal growth performance, feeding behavior, and animal health

**Figure 2:**
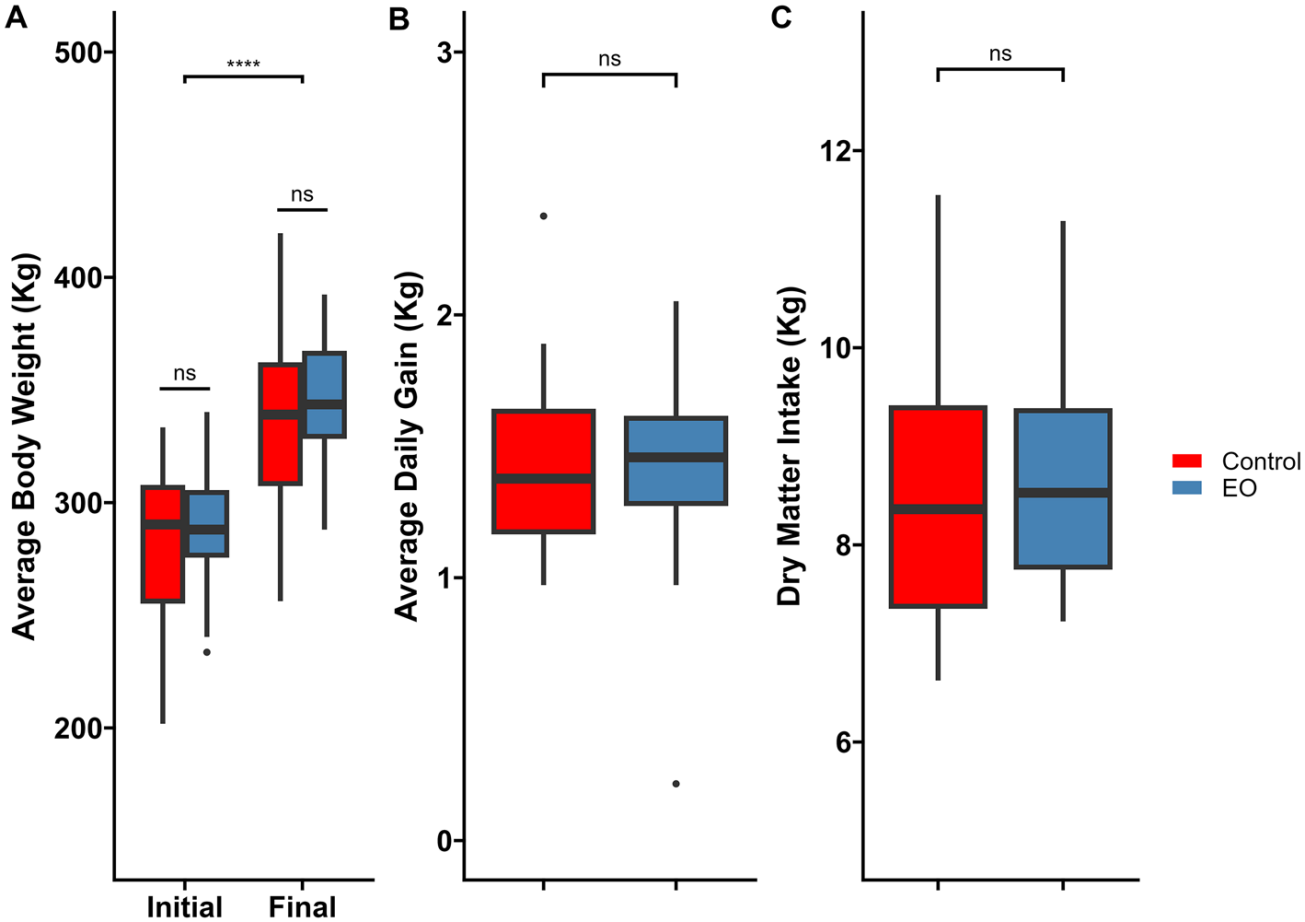
Performance parameters of beef steer calves including: (**A**) initial and final average body weight (kg), (**B**) Average daily gain (kg), and (**C**) dry matter intake (kg) up to 42 days post-intranasal essential oils (EO) or phosphate buffered saline (control) spray application. NS, no significant difference between treatments (*P* > 0.05); **** = *P* < 0.0001.

Calves that received a single dose of intranasal EO spray had similar (*P* = 0.43) initial (d-1) and final (d-42) BW when compared to control calves (Fig. 2). Additionally, there were no differences in ADG (*P* = 0.36) or DMI (*P* = 0.53) between the two groups (Fig. 2)**.** Although there were no significant differences between treatment groups regarding the average number of visits (*P* = 0.25), meals per day (*P* = 0.31) or eating rate (*P* ≥ 0.120), calves in the EO group spent a longer time at the bunk (*P* ≤ 0.05) compared to control calves (Table 1). Overall, all calves remained healthy and did not exhibit any clinical signs indicative of BRD during the course of study.

**Table 1:** Overall feeding behavior of calves that received intranasal EO spray or phosphate buffered saline (control) over 42 days.

	Mean		SEM	95% CI		*p*-value
	EO	Control		EO	Control	
Event, per day						
Visits	17.8	20.2	2.04	11.2–24.6	17.5–23.0	0.2
Meals	7.4	7.8	0.39	6.1–8.6	7.2–8.3	0.31
Time eating, min						
Per visit	6.82	5.30	0.74	4.4–9.3	4.35–6.34	0.046
Per meal	12.33	10.24	0.94	9.3–15.5	9.1–11.6	0.033
Per day	90.48	79.34	5.25	73.4–108.1	72.6–86.7	0.041
Eating rate, kg						
Per visit	0.77	0.64	0.08	0.5–1.0	0.5–0.8	0.134
Per meal	1.44	1.29	0.10	1.1–1.8	1.2–1.4	0.120
Per min	0.15	0.14	0.02	0.1–0.2	0.1–0.2	0.656

### 16S rRNA sequencing summary

A total of 266 DNS samples were collected from all animals in the study, including two negative control samples consisting of DNS swabs exposed to the air in the same area as the regular samples were obtained, and that were processed the same way as all other DNS samples. After quality filtering and removal of non-bacterial sequences, the average number of sequences per sample was 12,6610 ± 3,335 (SEM), for a total of 49,880 bacterial ASVs. After removing singletons and rarefying to even depth, the total number of bacterial ASVs was 18,359, which were subsequently classified into 36 different phyla, 409 families, and 1,128 genera, across all 266 samples. Amplicon sequencing of the V3-V4 region of the 16S rRNA gene is reliable to identify taxa down to a genus level, however, it is not precise enough for species classification of ASVs.

### Effect of intranasal EO spray on nasopharyngeal microbiota

#### Community structure of the nasopharyngeal microbiota

**Figure 3:**
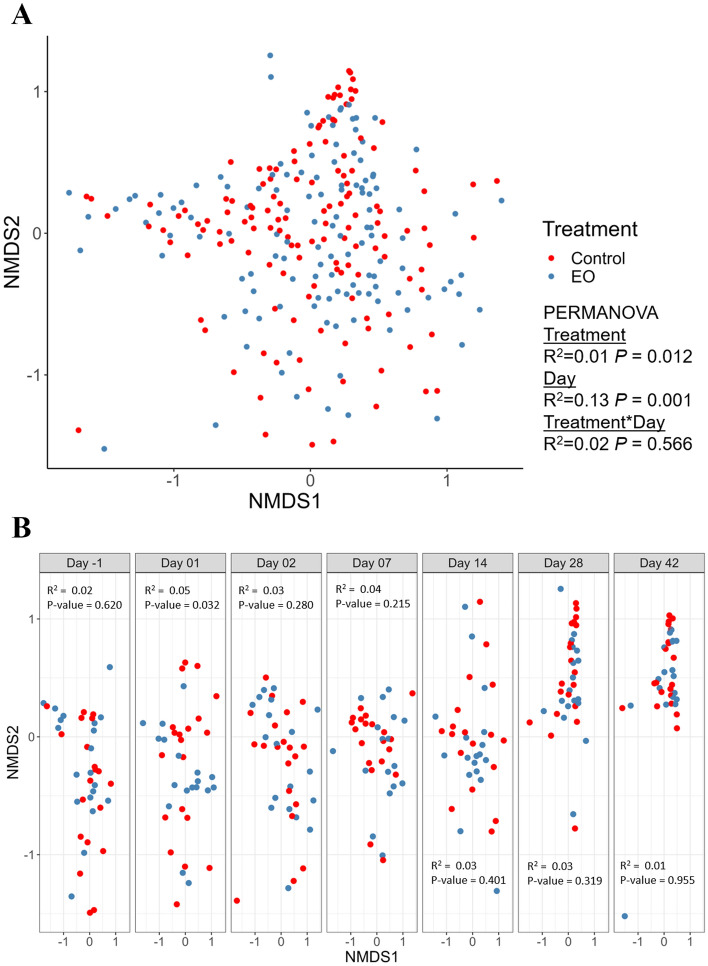
Non-metric multidimensional scaling (NMDS) plots of the Bray–Curtis dissimilarities for (**A**) nasopharyngeal microbiota in calves and (**B**) visualized by treatment and time. PERMANOVA R^2^ and *P*-values are indicated on the plots.

**Figure 4:**
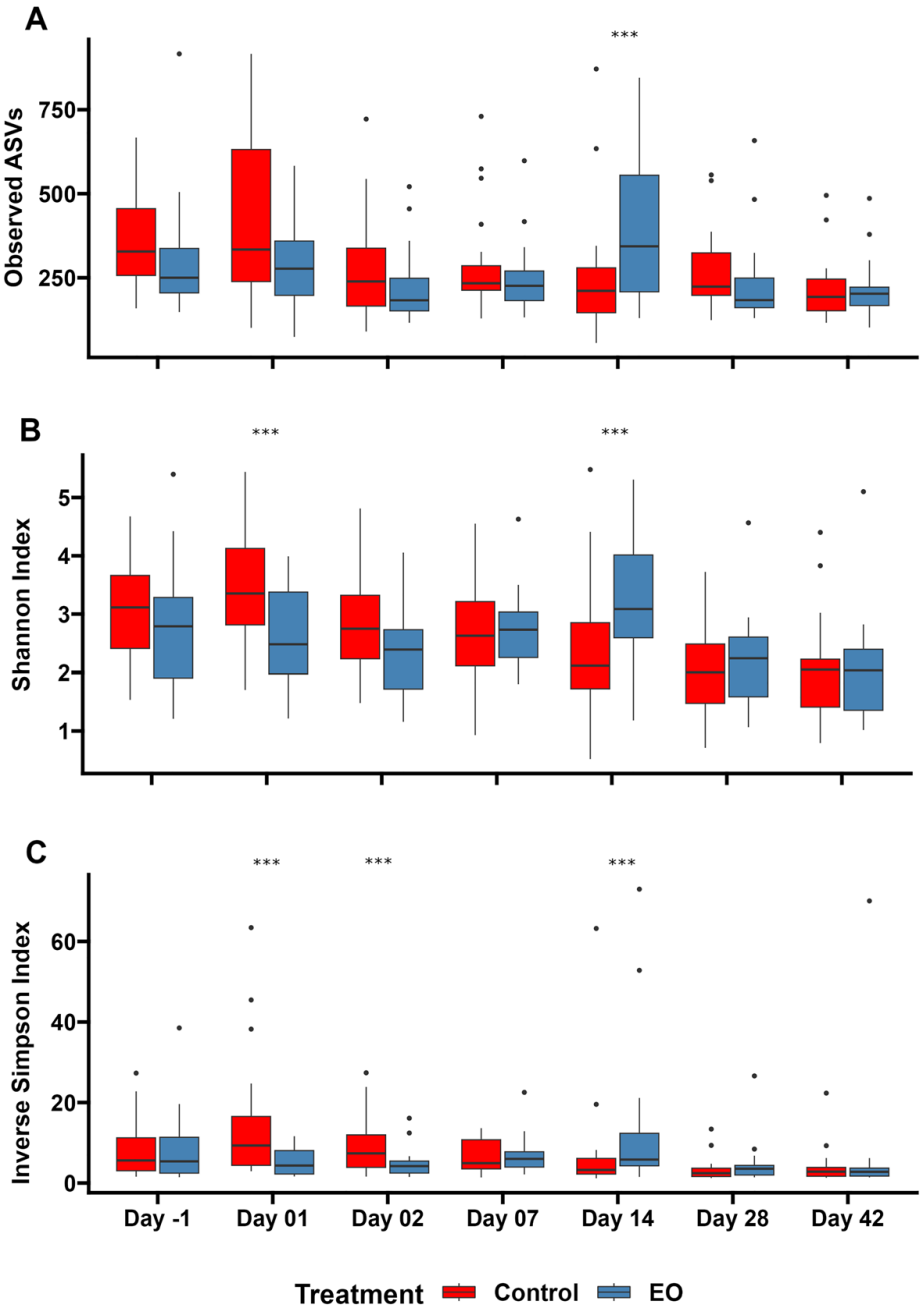
Boxplots of the alpha diversity measures of the nasopharyngeal microbiota in calves treated with or without essential oils (EO) by sampling time. On day 0, calves were treated with intranasal EO spray or phosphate buffered saline (control) (n = 20 per group). (**A**) observed amplicon sequence variants (ASVs), (**B**) Shannon diversity index, and (**C**) inverse Simpson diversity index. *** indicates significance at *P*-value ≤ 0.05.

The overall community structure of nasopharyngeal microbiota differed between the EO and control groups, although the treatment effect was relatively weak (PERMANOVA: R^2^ = 0.01, *P* = 0.02) (Fig. 3). The community structure was different between treatment groups (PERMANOVA: R^2^ = 0.05, *P* = 0.03) on d1; however, no differences were observed (*P* > 0.215) for the other sampling days (Fig. 3). Microbial community richness (observed ASVs) and diversity (Shannon and inverse Simpson indices) were significantly affected by EO treatment. During the first 24–48 h after EO treatment administration (d1 and d2), the Shannon and inverse Simpson indices were higher for the control group on (*P* ≤ 0.017). This effect was not persistent throughout the following days, however, on d14, the number of observed ASVs, and the Shannon and inverse Simpson indices were significantly higher for the EO group (*P* ≤ 0.03) (Fig. 4).

#### Composition of the nasopharyngeal microbiota

**Figure 5:**
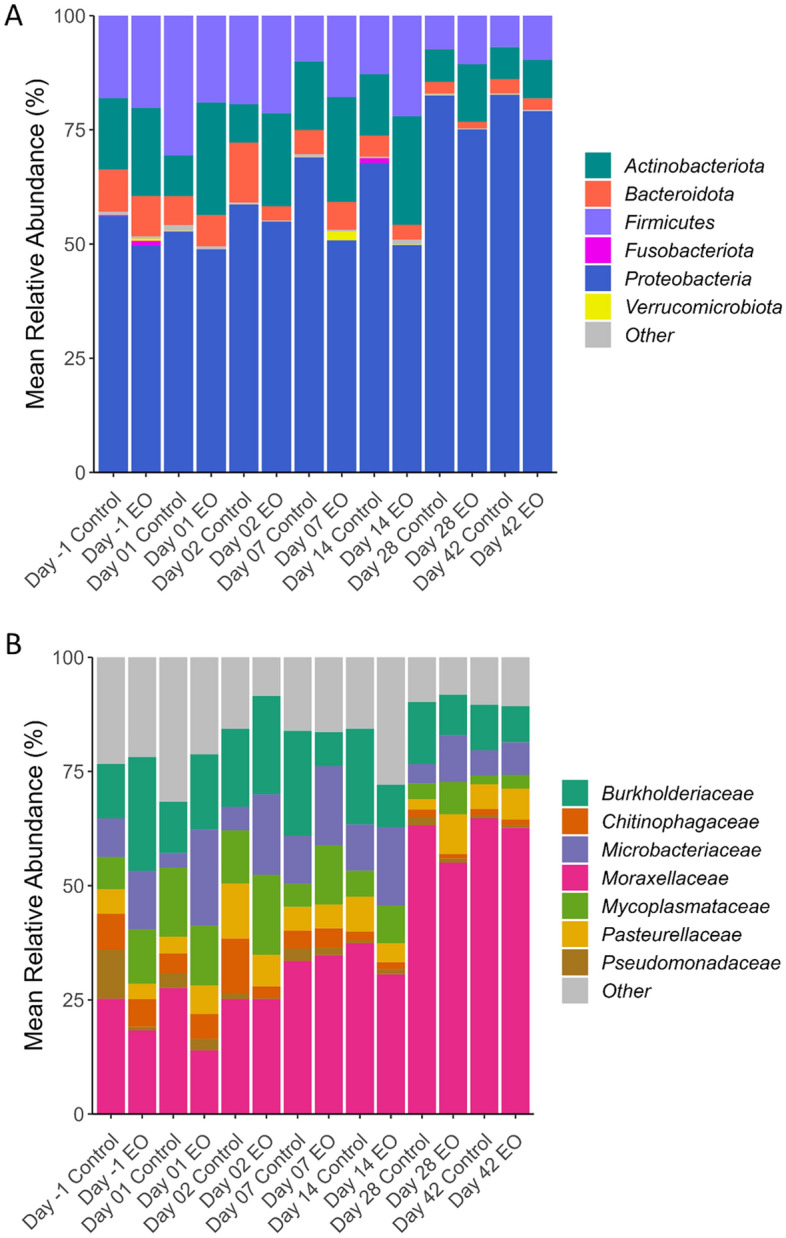
Relative abundance of most relatively abundant bacterial phyla (**A**) and families (**B**) in the nasopharyngeal microbiota of calves treated with or without essential oils (EO). On day 0, calves were treated with an intranasal (EO) spray or phosphate buffered saline (control) (n = 20 per group).

Of the 36 different phyla identified across all samples, the relatively most abundant were *Proteobacteria* (62.8%), *Firmicutes* (16.1%), *Actinobacteriota* (14.7%), and *Bacteroidota* (5.5%) (Fig. 5). The relative abundance of *Firmicutes* was lower in EO calves on d1, however, this phylum became more abundant in EO calves 2 weeks post EO treatment (*P* < 0.05). The EO group had a reduced relative abundance of *Proteobacteria* and *Bacteroidota* (d2 and 14) but a greater relative abundance of *Actinobacteriota* on d14 (*P* < 0.05). The most relatively abundant families overall were *Moraxellaceae* (36.9%), *Burkholderiaceae* (14.7%), *Microbacteriaceae* (10.6%), *Mycoplasmataceae* (8.8%) and *Pasteurellaceae* (5.9%) (Fig. 5).

**Figure 6:**
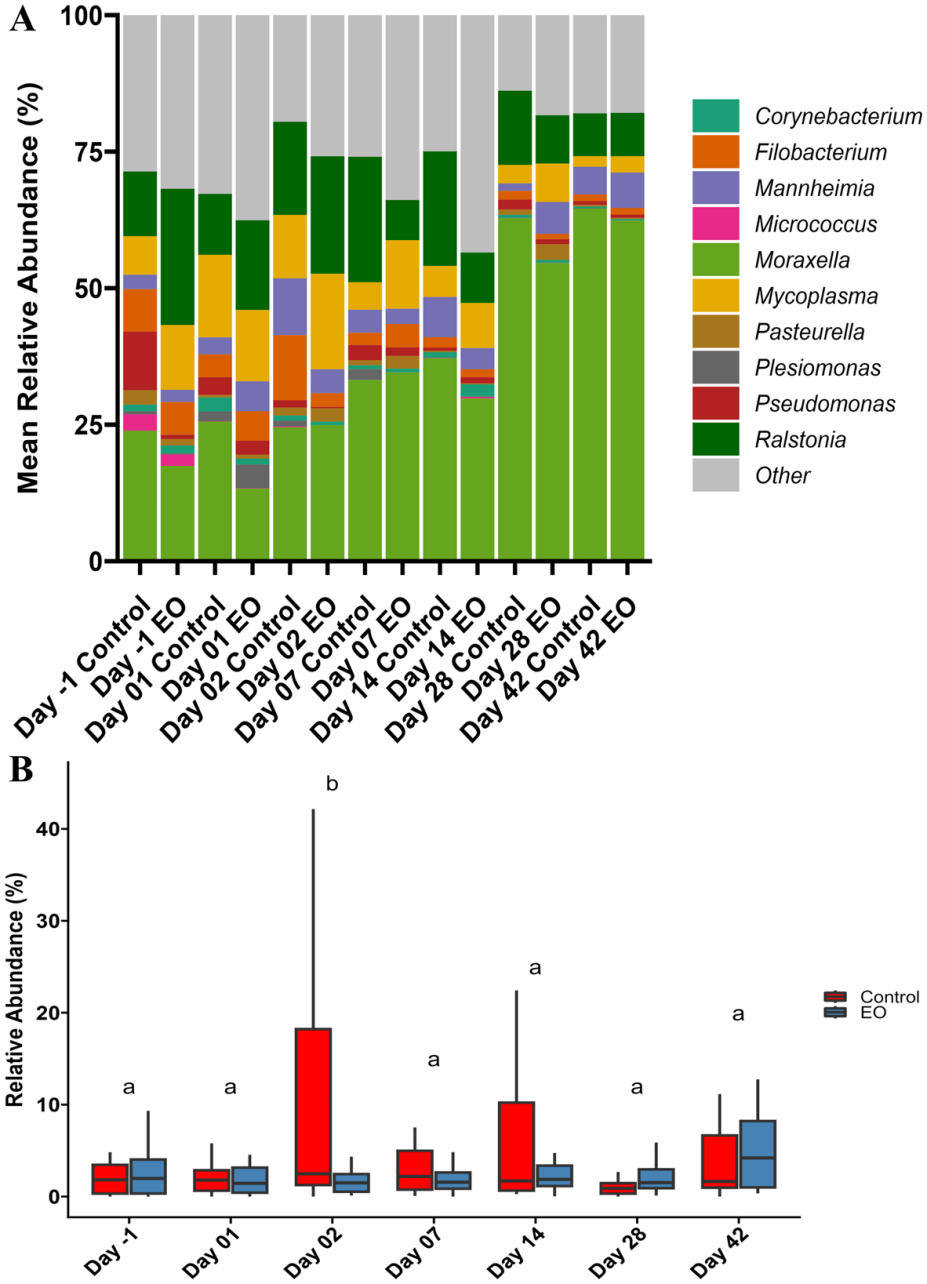
Stacked bar chart (**A**) showing the relative abundance of 10 most relatively abundant bacterial genera and box plots (**B**) showing the relative abundance of the *Mannheimia* genus. On day 0, calves were treated with an intranasal essential oil (EO) spray or phosphate buffered saline (control) (n = 20 per group). Different lowercase letters within each sampling time indicate significantly different means (*P* < 0.05).

The most abundant genera observed were *Moraxella* (36.28%), *Ralstonia* (14.52%), *Mycoplasma* (8.77%), *Mannheimia* (4.65%), *Filobacterium* (3.83%), *Pseudomonas* (2.10%), and *Pasteurella* (1.21%) (Fig. 6). *Mannheimia* (4.34% vs. 10.4%) (Fig. 6) and *Filobacterium *(2.53% vs. 11.9%) became less abundant (*P* < 0.05) on d2 in EO calves. In control calves, the relative abundance of *Mannheimia* increased by 3.9-fold (*P* = 0.03) from d-1 (24 h pre-EO treatment) to d2 (from 2.66% to 10.4%). However, this increase was not detected in calves that received intranasal EO on d0 (d0 = 2.23% vs. d2 = 4.34%; *P* > 0.05). The relative abundance of *Corynebacterium* decreased (*P* < 0.05) in EO calves 24 h post-treatment (d1; 1.06% vs. 2.58%). The overall relative abundance of *Pasteurella* did not differ between the EO and control groups (*P* > 0.05) (Fig. 6) and the relative abundance of *Histophilus* was too low (0.02%) to assess any EO effect.

### Effects of intranasal EO spray on the ecological networks of the nasopharyngeal microbiota

**Figure 7:**
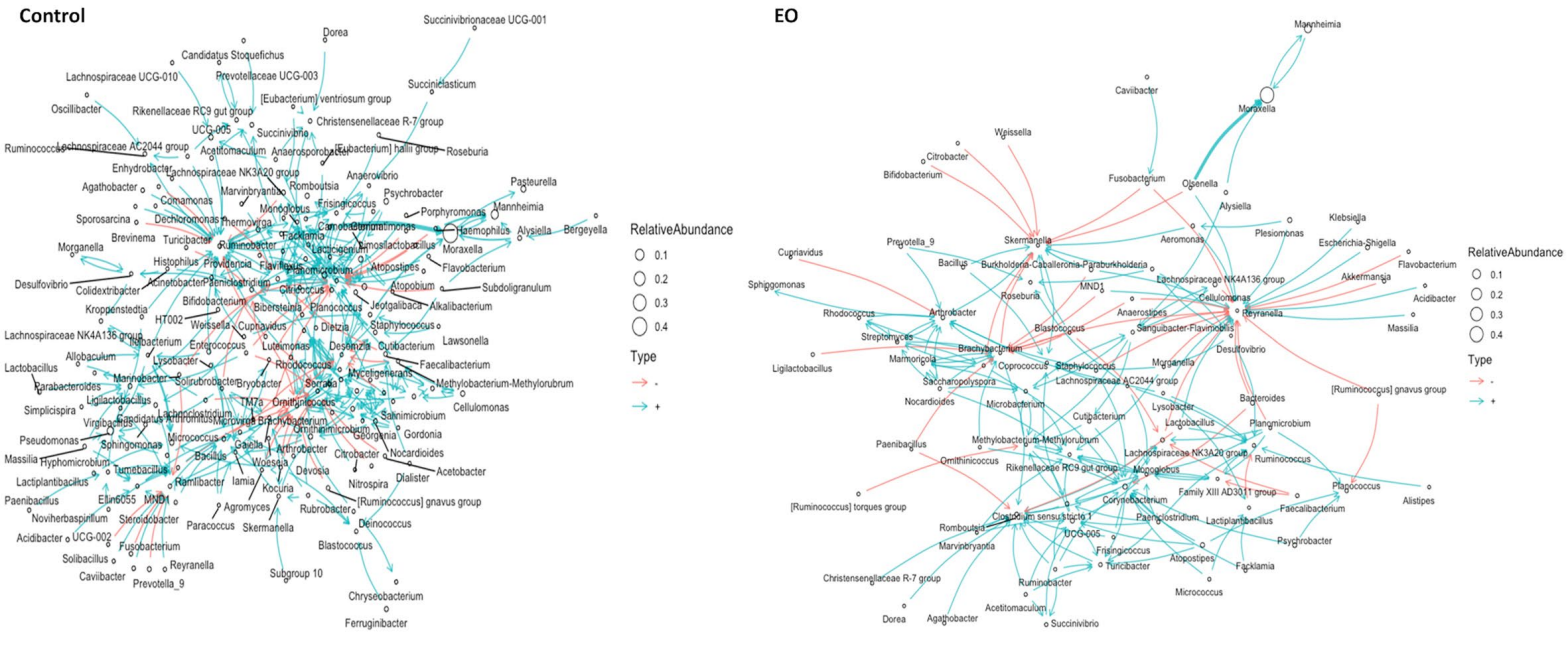
Ecological network of observed bacterial genera in nasopharyngeal samples of calves collected over the course of 42 days. On day 0, calves were treated with an intranasal essential oil (EO) spray or phosphate buffered saline (control) (n = 20 per group).

To evaluate the impact of EO on the overall microbial interactions of the nasopharyngeal microbiota, ecological network modeling was used to analyze the interaction of all genera. As shown in the network plots (Fig. 7), calves receiving EO spray had a distinct interaction network structure as compared to control calves receiving PBS. Compared to the network structure of the nasopharyngeal microbiota observed in control calves, the complexity of the interaction network of nasopharyngeal microbiota from EO calves was reduced with a 50% fewer number of genera remained in the model displaying positive or negative interactions with each other. Despite having a less intense interaction network structure, the total number of hubs connecting the interactions between genera and the number of genera with negative interactions were greater in EO group than that of control group. Overall, these results indicate that the EO treatment altered the interaction network among the EO microbial community by reducing the overall interaction intensity and influencing the dynamics and directions of the genera-genera interactions.

### Effects of intranasal EO spray on total bacterial abundance in the nasopharynx

**Figure 8:**
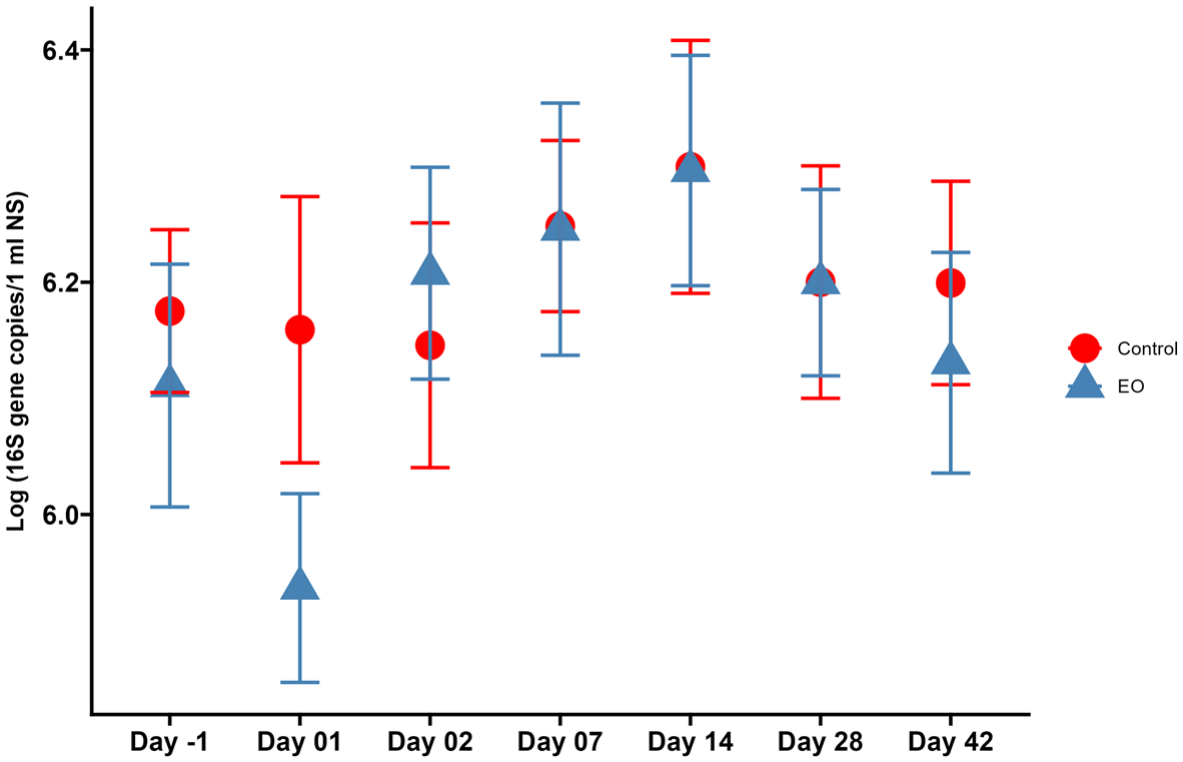
Total bacteria in the nasopharynx of beef steer calves (in log_10_), estimated by qPCR of the 16S rRNA gene between the essential oils (EO) and control treatment groups, over the 42-day sampling period. On day 0, calves were treated with an intranasal EO spray or phosphate buffered saline (control) (n = 20 per group).

Bacterial 16S rRNA gene copy numbers estimated by qPCR were not different between the EO and control groups (*P* = 0.45), and across sampling days (*P* ≥ 0.240) (Fig. 8). Overall bacterial concentration per nasopharyngeal swab, measured by the log_10_ of 16S rRNA sequences, ranged from 5.94 ± 0.08 (d1) to 6.30 ± 0.10 (d14) for the EO group, and from 6.15 ± 0.11 (d2) to 6.30 ± 0.11 among the control group.

### Effect of intranasal EO spray on BRD pathogen prevalence determined by culturing

**Figure 9:**
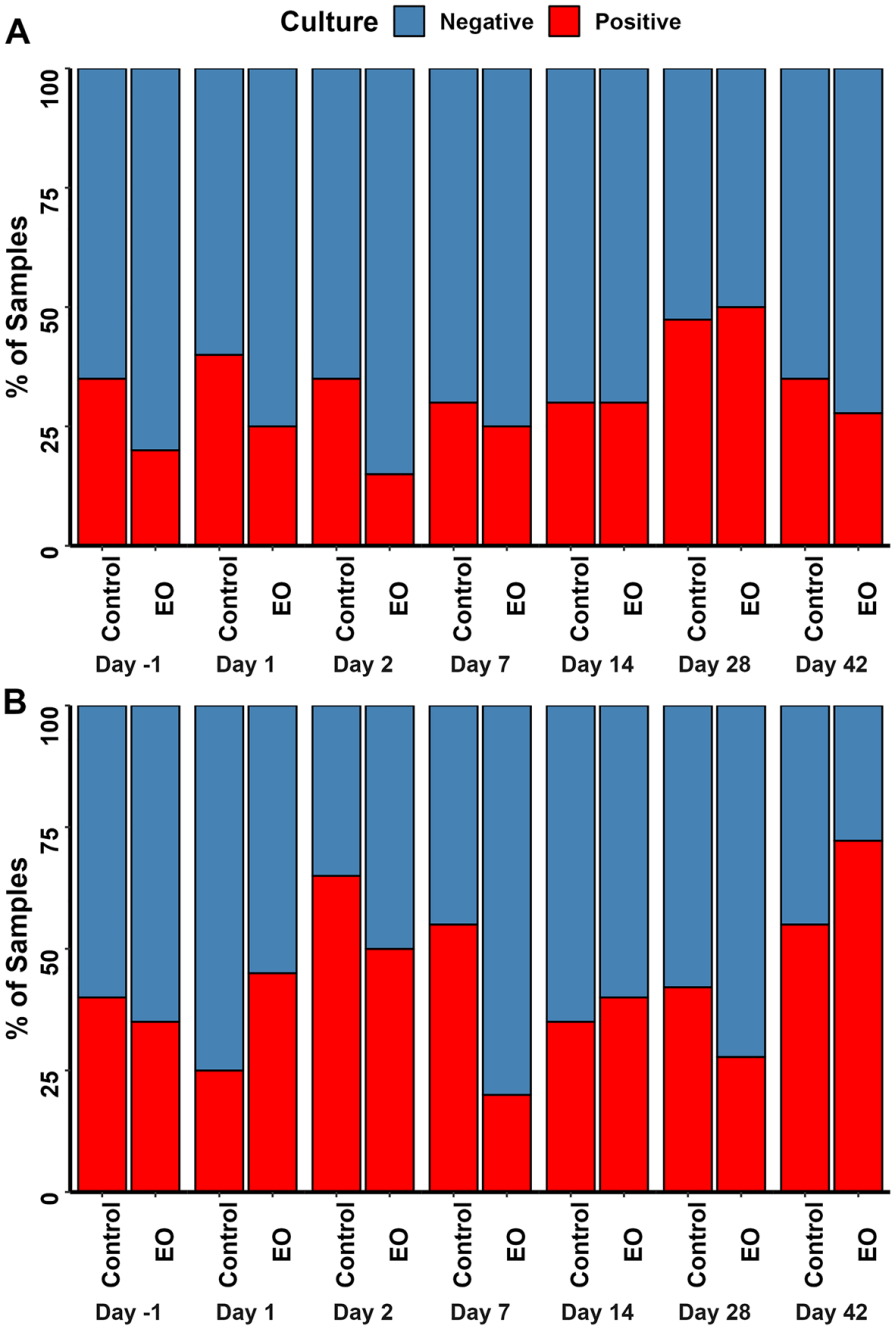
Prevalence of the bovine respiratory disease (BRD)-associated pathogens (**A**) *Mannheimia haemolytica* and (**B**) *Pasteurella multocida* in the nasopharynx of cattle over the course of 42 days, determined by culturing the nasopharyngeal swabs. On day 0, calves were treated with an intranasal essential oils (EO) or phosphate buffered saline (control) spray (n = 20 per group). * = significant difference between treatments (*P* < 0.05).

Nasopharyngeal swab samples were plated on blood agar supplemented with bacitracin and colonies with morphologies similar to *M. haemolytica*, *P. multocida*, or *H. somni* were isolated and identified by nearly full-length 16S rRNA gene sequencing. A total of 119 and 87 DNS samples were culture positive for *M. haemolytica and P. multocida*, respectively (Fig. 9). A chi-square test showed that *M. haemolytica* prevalence was greater (55% vs. 20%; *P* = 0.05) in the control calves compared with the EO calves on d7. The prevalence of *P. multocida* did not differ between the two groups at any sampling day (*P* > 0.05). Given that only seven samples were positive for *H. somni*, the effect of EO on the prevalence of this species was not assessed.

### Effect of intranasal EO spray on hemogram

Overall, blood hemogram parameters evaluated through a CBC (Table 2) were not affected by EO treatment (*P* > 0.08). The overall concentration of fibrinogen tended to be increased in EO calves (*P* = 0.08) while its concentration remained within the normal range of 300–700 mg/dL (Table 2). A significant time effect (*P* ≤ 0.02) was detected for many of the blood hemogram parameters including white blood cell count (WBC), lymphocytes, monocytes, eosinophils, red blood cell count (RBC), hemoglobin, mean corpuscular volume (MCV), mean corpuscular hemoglobin, and plasma protein (Fig. 1S).

**Table 2:** Hematologic variables of complete blood cell count (CBC) in beef steer calves up to 42 days post intranasal essential oils (EO) or phosphate buffered saline (control) spray application.

Parameter	Treatment		SEM	*P* values			Reference values*
	EO	Control		trt	day	trt*day	
WBC^1^ (K/µL)	9.53	8.94	0.180	0.399	0.005	0.126	4–12
Neutrophils (K/µL)	4.00	3.46	0.140	0.118	0.333	0.598	0.6–4
Lymphocytes (K/µL)	4.92	4.70	0.128	0.713	0.002	0.647	2.5–7.5
Monocytes (K/µL)	0.54	0.55	0.039	0.418	< 0.001	0.579	0.025–0.84
Eosinophils (K/µL)	0.41	0.32	0.044	0.406	0.016	0.548	0–2.4
Basophils (K/µL)	0.13	0.12	0.010	0.569	0.332	0.425	0–0.2
Neutrophils (%)	40.5	38.2	1.06	0.225	0.435	0.237	15–45
Lymphocytes (%)	50.4	52.5	1.093	0.385	0.005	0.296	45–75
Monocytes (%)	5.38	5.86	0.382	0.255	< 0.001	0.811	2–7
Eosinophils (%)	4.36	3.69	0.489	0.668	0.008	0.684	0–20
Basophils (%)	1.27	1.33	0.072	0.886	0.547	0.698	0–2
RBC^2^ (M/µL)	9.19	9.06	0.090	0.310	< 0.001	0.463	5–10
HGB^3^ (g/dL)	12.7	12.6	0.077	0.849	< 0.001	0.511	8–15
HCT^4^ (%)	36.3	36.4	0.276	0.791	0.059	0.956	24–46
PCV^5^ (%)	38.2	37.9	0.296	0.836	0.176	0.655	24–46
MCV^6^ (fL)	39.8	40.5	0.386	0.913	< 0.001	0.587	40–60
MCH^7^ (pg)	13.90	14.04	0.122	0.223	< 0.001	0.752	11–17
MCHC^8^ (g/dL)	35.0	34.7	0.135	0.373	0.243	0.961	30–36
PLT^9^ (K/µL)	481.8	486.6	16.713	0.641	0.757	0.557	100–800
Plasma Protein (g/dL)	7.60	7.58	0.042	0.788	< 0.001	0.629	7–8.5
Fibrinogen (mg/dL)	523.6	478.0	12.332	0.079	0.088	0.789	300–700

^1^White blood cell count.

^2^Red blood cell count.

^3^Hemoglobin.

^4^Hematocrit.

^5^Packed cell volume.

^6^Mean corpuscular volume.

^7^Mean corpuscular hemoglobin.

^8^Mean corpuscular hemoglobin concentration.

^9^Platelet count.

*Jones ML, Allison RW (2007).

## Discussion

Bovine respiratory disease is one of the most important infectious diseases affecting the feedlot cattle industry across North America. Its multifactorial epidemiology renders this disease challenging to control and prevent, resulting in producers frequently relying on metaphylactic antibiotic use^46^. This practice has been linked to the increase and spread of antibiotic-resistant BRD pathogens^47–49^. Therefore, there is an urgent need for the development of alternatives to metaphylaxis to mitigate BRD in feedlot cattle. We previously evaluated EOs for their in vitro antibacterial activities against BRD pathogens and commensal isolates originating from healthy feedlot cattle^31,32^. We identified five EOs that showed promise for inhibiting BRD pathogens while displaying minimal inhibitory effects on beneficial microbes including *Lactobacillus* strains with known probiotic potential against BRD pathogens^10,50^. In the present pilot study, we further evaluated the efficacy of these selected EOs on the nasopharyngeal microbiota, respiratory pathogens, animal performance, and feeding behavior when administrated intranasally to finishing feedlot steers.

Overall, a single intranasal dose of EOs had no negative effects on animal performance (DMI, ADG) or feeding behavior. Although to the best of our knowledge, there are no reports regarding the effect of respiratory application of EOs on dietary intake in cattle, the results of some nutritional-based studies are consistent with our observation. For example, dairy cows ingesting mixed rations containing a mix of cinnamaldehyde, eugenol, and capsicum EOs for 21 days had similar feed intake and feeding behavior to cows receiving a diet without EOs^51^. Dietary inclusion of a microencapsulated blend of EOs (carvacrol, cinnamaldehyde, eugenol, and capsicum) with monensin for 7 months did not result in altered animal performance in beef steers^52^. In the present study, while the number of visits, meals, and overall eating rate remained unaffected by EOs, overall time spent at the feed bunk was greater for EO calves compared to control calves. Given that ADG, DMI, and overall eating rate were not statistically different between the two groups, the longer time spent at the bunk by EO calves is difficult to explain. However, it is possible that the EO calves may not have been eating all of the time and rather standing with their head inside of the bunk. It has been reported that inhalation of EO mixture comprised of ginger oil, thyme, peppermint, and cypress hill resulted in reduced mobility of mice^53^. Overall, these results suggest that a single intranasal dose of EO administration at feedlot arrival does not have a negative influence on animal performance or feeding behavior in finishing feedlot cattle.

Hemogram results indicated that intranasal EO administration did not influence the CBC over the course of 42 days. However, sampling time had significant effects on some of these blood parameters evaluated. To the best of our knowledge, this is the first study evaluating the effect of intranasal EO on hematology in bovine animals. Cruz and colleagues observed no effect of feeding corn substitution by glycerin and EOs on the red and white blood cells of crossbred bulls finished in a feedlot^54^. An altered RBC and WBC, plasma protein, and fibrinogen are indicative of altered health conditions including inflammation, bacterial infection, or other systemic process^55,56^. Blood cell counts, and more specifically neutrophils and leukocytes, have been reported to be elevated in the blood of beef steers (11 months old) in response to nasal challenge with *M. haemolytica*^57^. Taken together, the unaltered hemogram results in EO calves that was observed here suggest that intranasal EO application may not trigger an acute inflammatory or immune response in finishing feedlot cattle.

Intranasal EO application did not influence the total bacterial load in the nasopharynx of EO calves. All five EOs that were included in the intranasal spray have previously displayed strong inhibitory effects against the BRD pathogens *M. haemolytica*, *P. multocida* and *H. somni*^31^. Species within the *Mannheimia*, *Pasteurella* and *Histophilus* genera and other genera whose cell membrane structure and physiology are similar to these BRD-associated genera were expected to be inhibited by these EOs. Therefore, we anticipated that total bacterial abundance would be reduced in the nasopharynx of EO-treated calves compared to control calves. While the mean bacterial load was not different between EO and control calves, the lack of effect of EO treatment on total bacterial load might be due to the volatile nature of the EO, as well as the dose and concentration of the EOs used in the present study.

The 16S rRNA gene sequencing results revealed that the nasopharyngeal microbial community structure differed between EO and control calves on d1, although this effect was relatively weak. As such, this effect was not sustained over the course of the study and the nasopharyngeal microbiota was similar between the two groups on subsequent sampling days. At the beginning of the study, 24 to 48 h post-EO application, there was higher microbial diversity observed in the control calves, which was indicated by the higher inverse Simpson index on d1 and d2 and the larger Shannon diversity index on d1. On d14, microbial richness and diversity were significantly higher in the EO group; however, this was not sustained over the following days (d28 and 42). Similar patterns of alpha diversity were observed in the nasopharyngeal microbiota in our previous study where we compared the effects of intranasal bacterial therapeutics (BTs) and metaphylactic antibiotic on the nasopharyngeal microbiota of recently weaned beef calves^50^. In this study, the calves received a single dose of intranasal BT comprised of 6 *Lactobacillus* strains had lower microbial richness and Shannon diversity index as compared to control calves that received intranasal PBS. Calves that received metaphylactic antibiotic tulathromycin, however, had greater microbial richness and diversity relative to both BT and control groups over the course of 42 days^50^. Immediate reduction of the microbial richness and diversity observed in EO calves followed a similar pattern observed in nasopharyngeal microbiota of BT calves while it contrasts with the pattern induced by the administration of tulathromycin. This observation coupled with the positive association of higher bacterial diversity and richness with pneumococcal disease status of children^58^ indicate that EO associated changes taken place in the alpha diversity of nasopharyngeal microbiota following immediately after EO treatment may be beneficial to bovine respiratory health. However, the increased microbial richness and diversity observed in EO calves on d14 was difficult to explain, and this change resembles the changes observed in the nasopharyngeal microbiota of calves following metaphylactic antibiotic treatment. Future studies to confirm the positive/negative association of increased microbial diversity and richness with respiratory health in cattle are warranted. Additionally, some compositional changes at the phylum and genus levels were detected for the first 48 h post EO treatment. One such change included a reduction in the relative abundance of the BRD-associated genus *Mannheimia* within the first two days of EO application.

Active interactions between different microbial species are important for maintaining the stability and functional features of the gut^59^ and respiratory microbiota^50^. Intensive interactions and multispecies with balanced positive (cooperation) and negative (competition) interconnectivity are positively associated with the functional activities and stability of a microbiota^60–64^. In the present study, EO treatment resulted in reduced interaction intensity between bacterial species within the nasopharyngeal microbiota. Compared to the control group, in which the interaction network structure was characterized by more positive interaction than negative, the network structure in EO group was built on equal proportion of positive and negative interactions between the bacterial species. Amat and colleagues also evaluated the impact of intranasal BT and antibiotic tulathromycin administration on the interaction network structure of nasopharyngeal microbiota in weaned beef calves^50^. The BT treatment intensified interactions among the genera while antibiotic administration resulted in near collapse of interaction network with only two dozen genera remained in the network model and interactions between them were limited to cooperation only^50^. Although the extend of diminished species-species interactions observed in nasopharyngeal microbiota of calves that received EO spray was not as severe as that of calves that received antibiotic, overall reduction of the interactions between bacterial species in EO calves was contrasting to that observed in BT calves. The comparison of the effects on network structure of nasopharyngeal microbiota between EO, BT and antibiotic treatments highlights a similar effect of EO to antibiotic on the microbial interactions, although with less intensity. The network analysis of co-occurrence patterns in a microbial community could provide information on the interactions, characteristics, and affinities between members of a community, which leads to the identification of patterns. This adds to the body of knowledge that these compounds can influence microbial dynamics in diverse environments. Thus, future research is warranted to identify whether the EO induced alteration of microbial interaction has negative association with the stability of respiratory microbiome in cattle. Nonetheless, the alteration of network structure of nasopharyngeal microbiota by EO clearly indicates that a single intranasal dose of EO spray could modulate the upper respiratory microbiome of cattle.

The effects of EO spray on the microbial community structure and composition of the nasopharyngeal microbiota detected within the 48 h post-treatment indicate that modulation of the upper respiratory microbiota by EO can be achieved in feedlot cattle but multiple doses of intranasal EOs would likely be required to obtain prolonged EO-mediated microbiota modulation. Manipulation of the bovine respiratory microbiota has become a new target for improved pulmonary health and enhanced resistance against BRD in feedlot cattle as the respiratory microbiome mediates the immune response and colonization resistance against respiratory infections^3,11,50,65^. Thus, restoring homeostasis of the respiratory microbiota by selectively inhibiting pathogenic bacteria while promoting beneficial endogenous members holds potential to mitigate BRD^13^. Future research is therefore warranted to evaluate the impact of multiple doses of intranasal EO at feedlot entry on the nasopharyngeal microbiota structure, richness, and composition in beef cattle.

The EO spray was administrated into the deep nasopharynx of calves, and the highly vaporous nature of the EOs might have resulted in only short-term impacts on the bacterial community at the mucosal surface of nasopharynx. The failure to induce more prominent alterations in the nasopharyngeal microbiota of EO-treated calves in the present study might also have been due to the concentrations of the EO applied. Although the concentrations of each of the EO included in the intranasal spray was based on their MIC against BRD pathogens plus consideration of potential cytotoxicity to upper respiratory mucosal cells^31^, the effectiveness of EO at the MIC determined in vitro may not be representative of the in vivo effects on the entire microbial community in the upper respiratory tract.

It has been documented that the MIC of EO determined in vitro tends to be 2–100 fold smaller than that necessary for achieving the same microbial reduction in food matrices^66^. This can be associated with the physiochemical characteristics of the environment such as pH, lipid, and water content of where the EO was applied. High fat and lower pH environmental conditions may render EO from reaching and affecting the bacterial cell bilayer by dissolving EO^67–69^. Another possible factor for the limited observed alterations observed in the nasopharyngeal microbiota in EO-treated calves might be associated with the respiratory microbiota being stable and resilient against external manipulation. The EO spray was tested in low-risk calves who were not sourced from an auction market, comingled with other calves from different sources, or transported for a long distance, which are all key management practices that can disrupt respiratory microbiota stability and homeostasis^3,11^.

We observed that a single intranasal dose of EO prevented an increase in the relative abundance of *Mannheimia* spp. within the first 2 days of EO administration and that the prevalence of *M. haemolytica* as determined by culturing was lower in EO-treated calves compared to the control calves on d7. All five EOs tested in this study have shown strong inhibitory effects against *M. haemolytica* serotype 1 strains in both vapor^32^ and liquid^31^ phases in vitro. It is therefore not surprising to see the reduction of *Mannheimia* spp. in EO calves. However, the single intranasal dose of EO did not provide consistent protection against *M. haemolytica* within the first 2 weeks post-EO treatment. This again might be due to the dose and concentrations of the EO administrated. In addition, the calves used in this study were at low risk for BRD and thus the relative abundance and prevalence of *M. haemolytica* were not as high as newly weaned, comingled, and stressed calves, which could have limited the effect of EO on *M. haemolytica*. Despite the fact that all five EOs exhibited in vitro antimicrobial activity against *P. multocida,* the effect of intranasal EO on this pathogen was not detected in the present study. Since the overall *Histophilus* abundance was relatively low across all animals, the effect of EOs on *Histophilus* was not assessed.

In summary, a single dose of an intranasal EO spray comprised of ajowan, thyme, fennel, cinnamon leaf, and citronella altered community structure, composition, and diversity and interaction network structure of the nasopharyngeal microbiota in feedlot steers, with more of an obvious EO effect occurring within the first 24 to 48 h post-administration. Calves that received EO had a 3.9-fold lower relative abundance of *Mannheimia* on d2, and lower *Mannheimia* prevalence on d7 compared to calves that received only intranasal PBS. Animal performance, feeding behavior, and hematology parameters indicative of inflammatory and immune responses were not affected by the EO treatment. Overall, the results of this pilot study suggest the potential for use of intranasal EO to modulate the bovine respiratory microbiome and mitigate BRD in feedlot cattle as an alternative to antimicrobial metaphylaxis. Future research is warranted to further investigate the efficacy of multiple doses of intranasal EO on the bovine respiratory microbiome, BRD pathogens, and immune response in feedlot cattle that are at high risk for BRD.

### Supplementary Information

Supplementary Figure 1.

## Data Availability

Raw sequence data are available from the NCBI Sequence Read Archive under BioProject accession PRJNA974097, BioSample accession SAMN35165222- SAMN35166970, SRR accession SRR24652257- SRR24652823. Other data that supports the findings of this study are presented within the paper.
