# Supplementary material for: A single intranasal dose of essential oil spray confers modulation of the nasopharyngeal microbiota and short-term inhibition of Mannheimia in feedlot cattle: a pilot study
Source: Sci Rep. 2024 Jan 8;14:823. doi: 10.1038/s41598-023-50704-1 (PMC10774355; doi:10.1038/s41598-023-50704-1)
Supplement: Supplementary file 1 — Supplementary Figure 1. [file 41598_2023_50704_MOESM1_ESM.pdf]

Supplementary Information File

A Single Intranasal Dose of Essential Oil Spray Confers Modulation of the Nasopharyngeal Microbiota and Short-term Inhibition of *Mannheimia* in Feedlot Cattle: A Pilot Study

Gabriela Magossi[1], Kaycie Schmidt[1], Thomas Winders[3], Zachary Carlson[3], Devin B. Holman[2], Sarah Underdahl[3], Kendall Swanson[3], Samat Amat[1]

[1]*Department of Microbiological Sciences, North Dakota State University, Fargo, ND, 58108, USA*

[2]*Lacombe Research and Development Centre, Agriculture and Agri-Food Canada, 6000 C & E Trail, Lacombe, AB, T4L 1W1, Canada*

[3]*Department of Animal Sciences, North Dakota State University, Fargo, ND, 58102, USA*

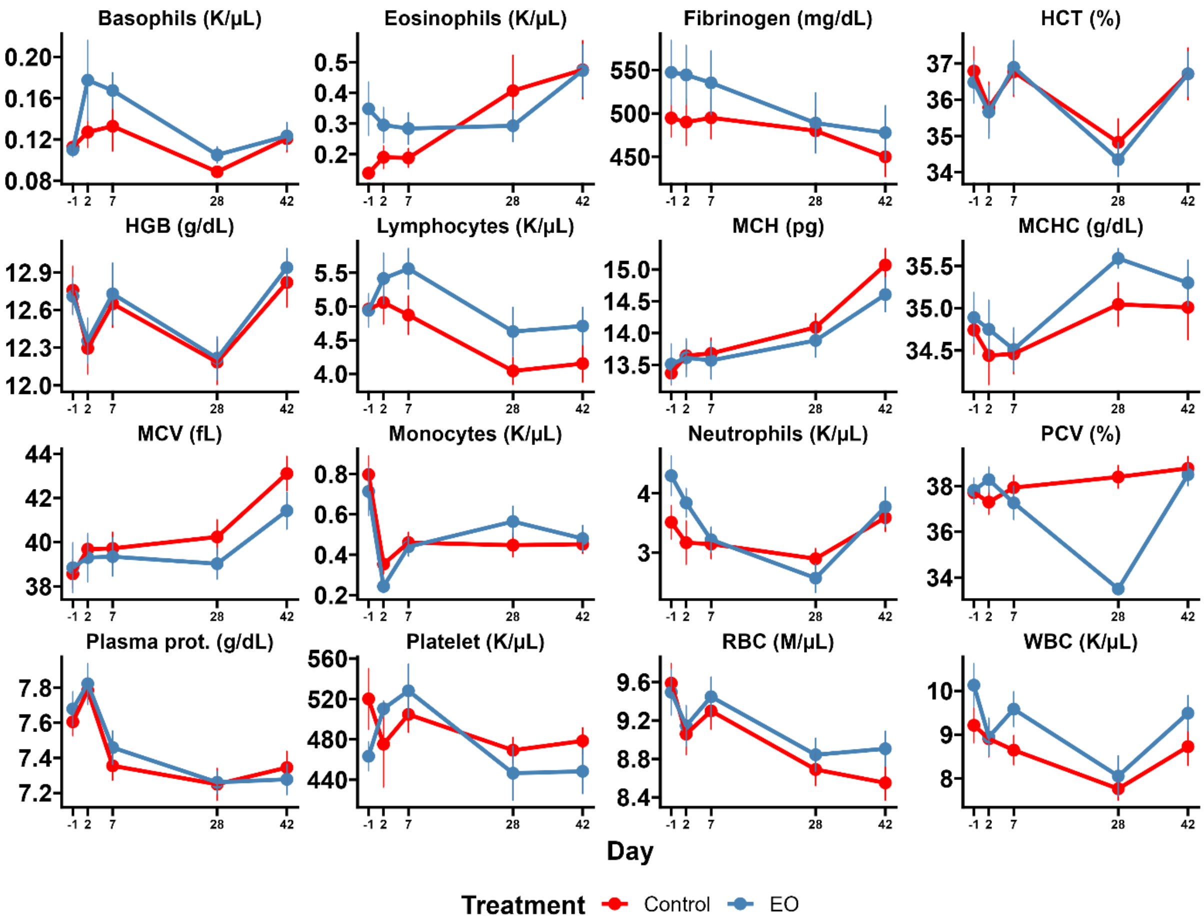

Supplementary Fig.S1. Complete blood cell counts from hematocrit of beef steer calves treated with an intranasal spray of essential oils (EO) or phosphate buffered saline (control) on day 0, across 42 sampling days (n = 20 per group).
